# 

**Published:** 1885-07

**Authors:** 


					﻿JUjLiY, 1885.
i
aaLkaa'aaa x a a ;
> _________________
►> i	__
► OLDSERlCS '
Vol. XXV.
. ssto
®'X-H© 5.
3&MLG O
fe MwW
Ikdigil^uO
^ijUr <^0URNAI§£v^
M fax et	e J
\r f /S' ^r-TTy—V J ^tvK?~-A	'f	?
•	W.F.WESTMOREUkND.tOj zs
LjM^k H.VM.MILLER.M.D.LL.D.®Lt z
| Hl^lfr	JAMES.A.GRAY. M£.^aZ
! r-ttfuBLlSHED By JAMES.P.HARRIS0N&CQ1W
Jiff _____________say LANTA, GA. &flKi
SUBSCRIPTION : $2.50 A YEAR.
				

